# Supplementary material for: Conditional chemoconnectomics (cCCTomics) as a strategy for efficient and conditional targeting of chemical transmission
Source: eLife. 2024 Apr 30;12:RP91927. doi: 10.7554/eLife.91927 (PMC11060718; doi:10.7554/eLife.91927)
Supplement: Figure 3—source data 3. [file elife-91927-fig3-data3.zip › Figure 3 source data3 part 2.zip!/Figure 3-Source data 3-part2/1060-9-CRISPResso_Report_q0_g4o36.zip!/CRISPResso_on_1060_10/9.Alleles_frequency_table_around_sgRNA_gtcctacgtctggtgggtca.pdf]

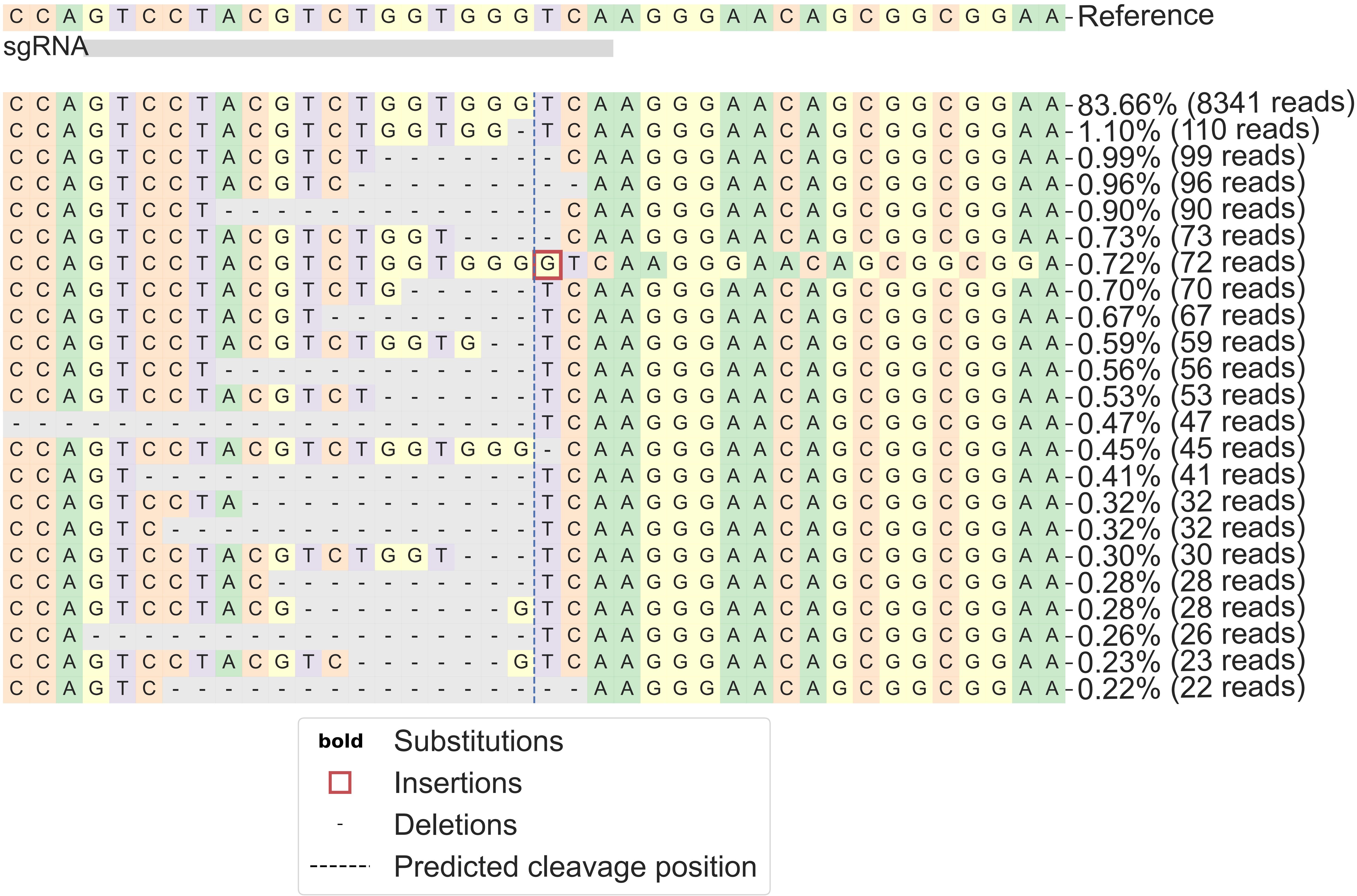
C C A G T C C T A C G T C T G G T G G G T C A A G G G A A C A G C G G C G G A A - Reference
sgRNA
83.66% (8341 reads)
1.10% (110 reads)
0.99% (99 reads)
0.96% (96 reads)
0.90% (90 reads)
0.73% (73 reads)
0.72% (72 reads)
0.70% (70 reads)
0.67% (67 reads)
0.59% (59 reads)
0.56% (56 reads)
0.53% (53 reads)
0.47% (47 reads)
0.45% (45 reads)
0.41% (41 reads)
0.32% (32 reads)
0.32% (32 reads)
0.30% (30 reads)
0.28% (28 reads)
0.28% (28 reads)
0.26% (26 reads)
0.23% (23 reads)
0.22% (22 reads)
bold Substitutions
Insertions
Deletions
Predicted cleavage position
